# The Nursing Supplement

**Published:** 1890-01-25

**Authors:** 


Being the Extra Nursing Supplement of "The Hospital" Newspaper.

All Contributions for this Supplement should be addressed to the Editor, The Hospital, 139-140, Salisbury Court, Fleet Street, London, E.C., and should have the word "Nursing" plainly written in left-hand top corner of the envelope.

### En Passant.

The Exhibition at Liverpool.—The nurse-dolls have had a very pleasant and very warm welcome at Liverpool. They were exhibited in the handsome board-room of the Hahnemann Hospital, which was specially decorated for the occasion; some lovely flowers being lent by the chairman. About sixteen more uniforms were added to the exhibition, including those of the Edinburgh Jubilee Institute, Northwich Victoria Infirmary, Devonshire-square Institution, and Liverpool Southern Hospital. The visitors to the show were very numerous, and the dolls were greatly admired.

Lay Nurses in Paris.—Once more the lay nurses of the Parisian hospitals have been attacked, and this time by Dr. Despres, who, though a staunch Republican in politics, is a great admirer of sisters of mercy. Taking the Charité Hospital, of which he is the head doctor, as an example, he declares that since the sisters were expelled two years ago, the patients have not been so well nursed and fed as before. From a financial point of view, the substitution of lay nurses for the sisters of mercy had been no less unsatisfactory. Formerly a sister received 200f. a year; each sister has, at the Charité Hospital, been replaced by three lay nurses, who receive salaries respectively of no less than 2,100f., 1,700f., and 1,500f. a year.

Sisterhoods and Influenza.—It is said that the nursing sisterhood of Saint Vincent, in France, have not suffered from the influenza, owing to certain sage rules of the founder. He discouraged asceticism, enjoined warm clothes and every reasonable comfort, because the sisters should regard themselves as fighting against the evil principles which cause suffering in the world and the combat with which needs bodily strength. They slept in good beds from nine at night to five in the morning, had good succulent diet, were free from care unless to do their duty, and were not to salute anyone by the way when outside of the convent on their errands of mercy. Salutations in cold weather were, said the reverend mother, one of the most fertile sources of throat and pulmonary disease. In fine weather they were a cause of loss of time, and took the mind from the object on which it should be set. We are glad to note this onslaught on the habit of kissing, a habit in which women indulge in a perfectly senseless manner.

Thousands of Pounds.—The actuary of the Pension Fund advises that if the Bonus Fund be increased to £40,000, or, better still, to £50,000, within a short time, there is reason to hope that adequate provision may be made for all the nurses of the British empire who avail themselves of the fund. This idea is excellent, and we propose to try, in co-operation with the council, to raise this money forthwith. We hope to be able to announce donations amounting to a thousand pounds every week till the required sum is raised. Contributions may be sent to the Secretary of the National Pension Fund for Nurses, 8, King-street, Cheapside, E.C., or to the Editor.

The First Thousand.

| | | | |
|---|---|---|---|
| Mr. W. H. Burns | £500 | 0 | 0 |
| Mr. T. H. Chance | 100 | 0 | 0 |
| Messrs. Messel and Co. | 100 | 0 | 0 |
| M. Van Raalte | 100 | 0 | 0 |
| Sir R. Wallace | 100 | 0 | 0 |
| Messrs. Ashurst, Morris, and Co. | 52 | 10 | 0 |
| Mr. H. L. Bischoffscheim | 25 | 0 | 0 |
| Mrs. Bischoffschiem | 25 | 0 | 0 |

Cottage Hospitals and the Pension Fund.
The committee of the Lyme Regis Cottage Hospital have resolved to pay half the matron's annual subscription to the Pension Fund. The subscription amounts to £6. 10s. a year, so that not much is asked from the committee, though much is given to the matron. We recommend this action to other cottage hospital committees as an example.

Kemerton Nursing Association.—The secretary writes: The above branch of the Rural Nursing Association, founded in Gloucestershire by Mrs. Malleson, has ended its first and experimental year with success, thanks to the happy result of an advertisement in your pages. Nurse Richardson, trained in Endell-street Maternity Hospital, came to work among us in consequence of this advertisement, and by her kindness and devotion has won the hearts of rich and poor, so that I am glad to say subscriptions are offered to us unasked, and we are able to begin with little balance at our bankers and a rise of salary to the nurse. Work among the sick poor in rural districts is very arduous, but of great value, and the gratitude of the sufferers who have been relieved is very touching.

Reductio ad Absurdum.—Truly if ever a poor society had to acknowledge that its schemes were become an absurdity, it was the council of the British Nurses' Association when they confessed that only *six* hospitals, and only three of these having over 100 beds, were willing to take part in a scheme of registration. We reprint the following paragraph from the report without further comment: "III.—In the matter of registration the Executive Committee issued a circular to the chairmen of all the hospitals in the kingdom engaged in the work of training nurses or midwives, explaining shortly the importance of the subject, and stating that a new body was about to be formed to undertake the work of registration, altogether distinct from the association, but upon which the association will be strongly represented by means of the members of its Executive Committee, offering each hospital the right to send one of its medical or nursing staff to represent its views upon the proposed council. As was expected, those hospitals which have been throughout opposed to registration declined to take any part in forwarding the scheme; others expressed their ignorance of the matter, and their consequent inability to take any action; others promised to give the subject further consideration, and the great majority made no official reply of any kind to the circular. Unofficially it has been stated that some hospitals are waiting to see the scheme in working order before they give their assistance to it; and others desire to know how the travelling and other expenses of their representatives are to be met if such are appointed. Finally, six hospitals—the York County Hospital, the Lincoln County Hospital, the Birkenhead Borough Hospital, the Halifax Infirmary, the Adelaide Hospital of Dublin, and the British Lying-in Hospital of London—appointed representatives (three medical men and three matrons). On December 6th a meeting of the new council was held, duly constituted itself, passed certain necessary by-laws and some preliminary regulations for registration, and appointed a representative registration board to carry out the scheme." The notion of the representatives of these six little hospitals solemnly sitting down and compounding a registration scheme for all the nurses of Great Britain is the most laughable farce we have heard of for a long time.

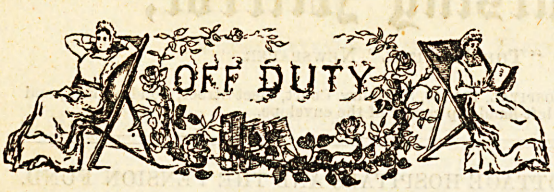

### Mr. Trantum's Hobby.

*(Concluded from page lxvi)*

So the days went by, and the two old men continued their work of building, but, alack! their materials were very poor, very flimsy. The little lad had no constitution to work upon. Although the salt sea breezes brought a colour into the wee white face, it was but a flush that faded woefully when they carried him indoors to The Bunk, and Darter Jane shook her head in a dismal manner that distracted her master and her father. Like all who would hold back one whom death beckons away, Mr. Trantum and Ben had come by swift degrees to be themselves so wrapped up in the little life they watched and tended that their hearts sank at a foreboding look. And Mr. Trantum had begun to love the child in a way that astounded himself. Choke them down, stamp upon them, if you will, there are tendrils rooted in the human heart with such vigour in them that while life remains—perhaps after, also, we all hope so, at least—they are ready to spring out, and twine round some object. The old naval officer congratulated himself often enough that neither wife nor child breathed to harass his even life with anxieties, and now it had suddenly come to pass that he found himself hanging with new-born anguish over a pining, dwining, little lad, Jack's boy—a mere nephew.

"I'm going to be a sailor, Ben," little Jack had said when he first came to The Bunk; "a sailor, just like my uncle, you know. I've made up my mind to go into the Navy; the Army's all very well, but, you see, there's the rest of the boys. They're sure to tumble in there as soon as they're old enough; besides, give me the sea, I love it, I do."

"That's right, my little hearty," said the gratified Ben, all aglow; "there ain't no man—tho' I says it, as should'nt—that comes up to a British tar. We'll fight for the Queen, God bless her, and for old England, while there's breath in our bodies. No land-lubber will beat us in a-doin' of our dooty. But you'll hev' to get hale, for there's a lot of larnin' to wade through afore you go afloat, I'm told."

"Yes, I suppose so," said Jack thoughtfully. "I can't go and be a middy right off. But I'm strong, Ben; oh, I'm all right," that was Jack's constant cry. He was ever "all right" and "much better"—a more cheery little invalid never suffered aches and troubles. But all the same, in spite of the sea-air cure and the long days on the sunny beach, the brave little man grew thinner, as Darter Jane noted—so did Mr. Trantum, with a pang. By-and-by, it came to pass there were days when even the two old sailors thought The Bunk was the best shelter for Jack.

"Drat them gusty nor'easters!" Ben would mutter savagely, but the weather was not to blame.

It was very gradual; it is nearly always mercifully so; and then came the morning when little Jack thought he would rather lie in bed an hour or two if Ben would come and whistle to him. It was such a rare delight to listen to Ben's whistling, he had a perfect *répertoire* of nautical tunes, through which he plodded in regular order, the child soon learning to pounce upon any attempt at deviation from it. After that morning, it began to be the daily custom for Ben to sit beside the little bed whistling, and shaping a fleet of tiny boats which sailed over the white counterpane, guided into port by the small feverish fingers.

Then it would be Mr. Trantum's turn to cry, "My watch now, Ben; off with you."

"Ay, ay, sir," and the old officer would sit and spin yarns or read out thrilling sea stories, pretending that he and Jack were very jolly, Darter Jane going in and out meanwhile in silence. For worlds the good soul would not have ventured to put her thoughts into words now, but she was not deceived.

"Perhaps a doctor could do something for the boy's breathing," at last said her master nervously.

"I don't hold with doctors myself, sir," said Darter Jane, "though I can't say but what they're useful in one way—they save reflections arter."

"Confound you! what do you mean?" ejaculated Mr. Trantum; but Jane had retired to calmly set about preparing barley-water.

A doctor came, saw, and was beaten. He frankly said he could do nothing, and, with a groan, the old sailor bowed his head.

"The word has come, Ben; my little lad is to set sail, and God help me!"

"He will, sir; He will. The Great Commander knows best. If so be as it was you, sir, or me, sir, we'd nayther of us dispute the order." Thus, did the master and man help each other to bear the blow.

How fast came on the dreaded end; with what rapid strides it approached!

"I don't expect I'll go into the Navy, after all, uncle," said little Jack, wistfully; "somehow I don't feel so keen about it as I did. I'm always so tired, now, you see; and they wouldn't care much for a chap with no breath to climb. I believe I couldn't so much as cheer the Queen; see, here's her yacht coming," and the gay royal boat moved slowly over the counterpane. "Hooray!" the cheer was sadly weak; "there, that's all I could do, and her Majesty wouldn't hear it!"

"Shouldn't you like to see your mother, Jack?" The question was asked in a hoarse quaver, not that the little lad noticed. He was growing strangely inobservant of late.

"No, no, I don't think so. She couldn't very well come here. There's always such a lot of parties she's obliged to go to, you see." Jack spoke quite simply, as though parties were the bounden duties of a mother. "I don't care about anyone but you—and Ben—uncle."

"Little Jack, what should you do if you were ordered to take a long voyage, if supposing the Almighty commanded it?" The old sailor found a choking difficulty in shaping his meaning, but duty to the dying child must be done.

"It's all right, uncle, I know." Little Jack lifted his bright eyes from the royal yacht. "I'm quite ready when the word comes"; the boy spoke quietly and tranquilly while Mr. Trantum gasped. Then, it was no news to Jack. No! we cannot tell how, but the dying are not left in ignorance of their call. And so lovingly, so gently, the knowledge steals over them that there is no fear.

"I want Ben to come and whistle," said Jack, presently.

In the silent room sat the two old men on either side of the little bed; one fighting down a heart-break, the other bravely whistling gay sea ditties.

"Ben," suddenly interrupted little Jack, "I'm afraid I can't wait until you get through them all this morning. Please miss over a few, and let me have "Tom Bowling."

As the last notes of the famous old sea song died away, the tiny fingers relaxed their hold of the royal yacht, and little Jack had set sail. The word had come; he heard the voice of the Great Commander. That was enough—Jack was quite ready.

Winter storms and summer spells have passed over The Bunk. There is little alteration in the quiet seaside spot; only to strangers is always pointed out a small, new house, something like a miniature barrack.

"That's Mr. Trantum's hobby. Who is Mr. Trantum? Why, he's the old Navy gentlemen as lives at The Bunk; he raised that there hobby some years ago, and winter and summer, he has a matter of six little lads in it a-buildin' of 'em up, as his old sarvint-man, Ben Bromich, calls it. Happen you've seen Ben in the churchyard yonder. He goes there reg'lar to whistle beside the little grave of Mr. Trantum's nephew. Master and man was bound up in the little chap, and when they lost him they took on dreadful. But they brightened up a bit when this house was set a-going. No matter who the lads are, so long as they're weakly they're welcome; and when they are turned out hearty then others take their places. Yes, he's a rare, good old gentleman, he is, and that's his hobby."

So it is, but in his heart Mr. Trantum calls it, not his hobby, but his monument sacred to the memory of little Jack.

### The Nurses' Bookshelf.

### OBSTETRIC NURSING.*

This little handbook gives the methods carried out in the Edinburgh School of Medicine for the training of midwives, a school whose training has justly a very good reputation, and purports to present in a portable and accessible form as much theoretical knowledge as is required for the diploma of midwife, and therefore, as the preface sets forth, the volume is very simple, "and, while endeavouring to supply the monthly nurse with all the necessary instruction, we have intentionally omitted much scientific detail, and confined ourselves to practical and essential facts." How thoroughly this purpose has been carried out as a whole we shall now proceed to show by an analysis of the contents of the book.

We must, however, take exception to the very bald and dry outline of physiology with which the book opens. Why should physiology for nurses be made so very uninteresting, as it almost invariably is? It is perfectly true that a nurse should have some knowledge of the structure of the body; but let her knowledge be that of the body clothed in flesh and muscle, sentient, and full of life, not of the body as composed of dry bones only. Moreover, the diagram of the circulation of the blood, to any nurse who gets her sole knowledge of physiology from this introduction, is so very diagrammatic that it merely suggests the idea to the uninitiated mind that the impure blood occupies the whole of one side of the body, and the pure blood the whole of the other. It seems a pity, therefore, that the authors did not give a larger diagram, coloured red and blue, showing the circulation as arranged by Nature in a non-diagrammatic representation.

As regards the author's directions for taking temperatures, we would remark two or three points which vary slightly from what is usually taught: 1deg. normal temperature is here stated to be 98·6deg.—this is a very slight variation, still, as 98·4deg. is the orthodox normal temperature as taught to all nurses who have not the privilege of being trained in the Edinburgh School, it seems a pity that nurses should be unnecessarily confused even on the smallest point because "doctors differ."

Another direction to the nurse in connection with the taking of temperature is "previous to introducing the thermometer the mercury should be shaken down below 98deg." This is a vague direction for the nurse who hopes to get all the necessary theoretical instruction from this little handbook, and who should have been told to shake down the index below 96deg., as there are such things as very subnormal temperatures, which have their significance, as the authors admit afterwards, though they omit to give the necessary directions how to discover such a temperature. The directions to the nurse to dry the axilla previous to inserting the thermometer are excellent, and might with advantage be carried out more systematically by every nurse, whether obstetric or otherwise. The history of the vagaries of temperature as noted by an experienced nurse would be most instructive and interesting; including, as it would, the differences in the temperature of the same patient when taken by different thermometers, even when done most conscientiously, and the temperature of a hysterical patient, which may run up to 103deg. or 104deg., even though she be about all day, and eating with an excellent appetite. In view of these experiences, we believe the only way to arrive at the truth is to insist on having a temperature that presents anything abnormal taken with two or three different thermometers.

The authors, thoroughly appreciating the importance of antiseptics, devote a whole chapter to their consideration, and explain fully and well their great importance. The two antiseptics most recommended are carbolic acid and corrosive sublimate or perchloride of mercury. The most useful strengths of each are carefully given, and attention called to perchloride of mercury pellets, which can be procured, and one of which, dissolved in a specified amount of water, gives a solution of the strength of 1 in 2,000, the convenience and portability of which should recommend them to the monthly nurse.

Considering, however, the very absorbent nature of the mucous membrane to which the solutions are to be applied, and how poisonous both of them are, it is a pity the authors have not given briefly the chief symptoms of poisoning by each, as some patients are very susceptible. For the benefit of nurses in general, we would remind them that in using perchloride of mercury soreness of the teeth and gums and increased salivation should be at once reported to the medical attendant, and if carbolic be used in large quantities any green appearance in the urine should be reported. The mucous membrane is thus described: "The cavity of the uterus is lined by mucous membrane very similar to that which lines several of the cavities of the human body; such as the mouth, stomach, nose, etc. The mucous membrane of the uterus, like other mucous membranes, secretes a small quantity of fluid. Mucous membranes, besides secreting mucus, are capable of sucking up or absorbing other fluids or solids which may be applied to them. For instance, the mucous membrane of the stomach and bowels absorbs nourishment for the body from the food we eat. The mucous membrane of the uterus is capable also of absorbing substances very rapidly, especially after labour. This cannot be insisted on too strongly, keeping in mind what has been said about puerperal fever, and the absorption of poisonous materials into the blood."

We are glad to see antiseptic cotton wools of various kinds recommended instead of sponges, and would be glad to see even an extended use of pads of wool or wood-wool wadding covered with sublimate gauze, so that these should entirely supersede towels of all kinds. Thus would be removed a source of danger to the patient, and of anxiety to the nurse.

Dr. Halliday Croom's "Rules to be Strictly Observed by Nurses" are excellent, and all the sixteen cannot be too carefully followed. In Rule 1, about washing and disinfecting her hands, since this handbook is for inexperienced nurses, it might also be well to advise her to take great care lest her hands get chapped, and she thus run risk of infecting herself in any way. Probably no one knows as well as a nurse how terribly carbolic acid does chap the hands, and we think every young nurse should take care of her hands from the first, and remember that "prevention is better than cure," especially when such simple things as glycerine or vaseline rubbed in at bedtime will prevent it. In addition to carefully scrubbing the nails, it is an excellent plan to fill them with thymol, or other approved antiseptic jelly or soap.

(*To be continued.*)

* "Handbook of Obstetric Nursing." By F. W. N. Haultain, M.D., and J. Haig Ferguson. Young J. Pentland.

### A Visit to Winchester.

Amongst the many invitations to Christmas entertainments which reached us was one specially enticing little green programme from the Royal Hants County Hospital. It was impossible to refuse such a pretty invitation, so on January 16th our representative took train from Waterloo to Winchester. It was a lovely day, and the sunshine was so warm it seemed impossible to believe that winter was still with us and Christmas not yet a month behind. Winchester is a delightful old town built on the side of a steep hill; the cathedral is full of interesting relics, and our army sisters might well gaze with awe at the long list of those soldiers who died in the Crimea, and whose names are here inscribed on different brasses. Sad to note that the deaths from cholera and fever were as numerous as those from wounds. The tattered colours which came through the various campaigns hang above the various memorials; themselves the best memorials of the gallant soldiers whose names are written below. Amongst other tombs are those of Hardicanute, William Rufus, Cardinal Beaufort, Jane Austen, and Francis Francis. "Into the night go one and all!"

Climbing up the steep High-street one passes without the city gate to where a monument stands to mark the spot where the country people placed provisions for the town when it was visited by the plague. The citizens were not permitted to come forth and spread the sickness, but the country neighbours brought food and left it within reach of the sore-stricken town. The Royal Hants County Hospital is on the very top of the hill, and commands a most extensive view over the town and over the country far beyond. The centre of the building is occupied by offices, etc., and on each side stretches a wing of three storeys—that to the left containing three wards, that to the right containing two wards and the chapel. The old hospital was down in the town and was founded in 1753, and supposed to be the first institution of the kind in England. The present building is only eighteen years old.

The first part of the Christmas entertainment took place in the men's ward, which had been cleared for the occasion, and where a stage had been erected. The front seats were occupied by the patients, all the women wearing blue or red cloaks and neat white caps. A few children were present and they wore scarlet cloaks and hoods of the most picturesque description. The sisters and nurses sat with the patients; behind was a large concourse of visitors which quite filled the large room. Mrs. Suckling played a lively overture on the piano, and then the curtain was rung up and the farce "Good for Nothing" was commenced. The acting was very good, Miss Jones-Bateman as "Nan" being loudly applauded. Afterwards the visitors adjourned to the accident ward, where a huge Christmas tree was flaring with numerous lights. Toys and crackers were on the tree, and round its foot were numerous parcels of warm clothing to be given to the patients. Mrs. Suckling distributed gifts to visitors and all, and also provided a most enjoyable tea. Downstairs a room was cleared and a drugget down, and there was a supper-table laid out which would have done credit to Gunter. The general belief was that the nurses were going to end up the evening with a dance. Amongst those present at the Christmas entertainment were the Countess of Airlie, Lady Frederick, Mr. and Mrs. Jones-Bateman, Colonel Heathcote, Colonel Eden, Colonel and Mrs. Bouverie Campbell, General Forrest, C.B., Dr. Graham, Dr. Harman, and many more friends of the hospital too numerous to mention. One and all congratulated Mrs. Suckling very heartily on her successful entertainment.

### Appointments.

[It is requested that successful candidates will send a copy of their applications and testimonials, with date of election, to The Editor, The Lodge, Porchester-square, W.]

Gibraltar Civil Hospital.—Miss Edith Mawe has been appointed sister-in-charge of this important hospital. Miss Mawe trained in the North in 1884, spent a year at St. Bartholomew's in 1886, and then gave two years to dispensing. Miss Mawe then took a school of cookery course, and subsequently became matron to an orphanage hospital. For the last five months she has been working in Mrs. B. Fenwick's private hospital. We congratulate Miss Mawe on having secured a post likely to bring into use all her many and various accomplishments.

Longton Cottage Hospital.—Miss Peck has been appointed matron of the Longton Cottage Hospital, for which appointment there were 54 candidates. Miss Peck trained at Addenbrooke's Hospital, Cambridge, for which she has a certificate. She has held the appointments of night superintendent at Salisbury Infirmary, matron of the Yeatman Hospital, Sherbourne, and matron of Bootle Borough Hospital, Liverpool. She was also *locum tenens* matron at Bristol Hospital for two months. Miss Peck is well spoken of by the medical practitioners and lay authorities of the institutions with which she has been connected, the committee at Bootle Hospital saying that too much could not be said of Miss Peck's fitness to preside over such an institution, both as a disciplinarian and as an economical housekeeper.

Stafford Infirmary.—Miss Mary Bettney has been appointed matron of this infirmary. Miss Bettney trained at the Children's Hospital, Pendlebury, and the Birmingham General Hospital, and was subsequently assistant matron at the Great Northern Hospital. Lately Miss Bettney has acted as temporary matron at the Jaffray Hospital. There were 43 applicants, but Miss Bettney had a large majority of votes, and our good wishes go with her to her new post, while we congratulate the committee on their choice.

### Death in our Ranks.

We regret to record the death, on January 9th, of Miss A. J. Fitzgerald, eldest daughter of the late Bishop of Killaloe, and superintendent of the City of Dublin Nursing Institution.

### Notices.

To Our Readers.—In view of certain changes which are imminent in consequence of the large and ever-increasing circulation of The Hospital, the Editor requests every matron, sister, or nurse who takes the paper regularly to send him notice of her name and address, to The Lodge, Porchester-square, London, W. Postcards may be used.

Burdett's Hospital Annual.—In reply to many enquiries, we beg to state that "Burdett's Hospital Annual" for 1890 is now in an active state of preparation. It cannot, however, be ready before the middle or end of March, as an attempt is being made to include the figures for 1890 in as many cases as possible. We understand that this Annual will form the basis for the distribution of the £2,236 under the *Tit Bits* hospital scheme.

Nurses' Note-Book.—All applications for this must be made to the Publisher of The Hospital, 139, Salisbury-court, E.C., *and not to the Editor*. Price 1s. 1d., post free. Payment must be made with order.

### Vacancies.

### Matrons.

| | |
|---|---|
| Nottingham Samaritan Hospital, £50. | East London Nursing Society, £75. |

### Night Superintendents.

| | |
|---|---|
| Bradford Infirmary, £30. | Salford Union Hope Infirmary, £27. |

### Nurses.

| | |
|---|---|
| Bath Royal United Hospital, £25. | Jersey Nurses' Institute, £25. |
| Blackburn Infirmary, £27. | Kent Nursing Institution. |
| Bournemouth Institute, £25. | Kingston Nurses' Home. |
| Brompton Cancer Hospital, £25. | Levick Institution, Paris. |
| Cardiff Infirmary. | Montreal, Canada, £37. |
| Central London Throat Hospital, £25. | Mutley Nursing Home. |
| Chelsea Infirmary, £18. | North-Eastern Hospital for Children, £20. |
| Croydon General Hospital. | Norwich Hospital. |
| Croydon Nurses' Institute, £20. | Nottingham Nursing Association, £25. |
| Deaconess House, Chester, £24. | Nursing Institution, 96, 97, 98, Wimpole-street, London, W., Mr. Wilson has several vacancies for thoroughly-trained nurses. |
| Dorset County Hospital. | Smallpox Hospital Ships, £24. |
| Dublin, Cork-street Hospital, £25. | Southport Infirmary, £25. |
| Eastbourne Home Hospital. | Workhouse Infirmary Association. |
| Exeter Hospital, £24. | York Home for Nurses. |
| Frome Nurses' Home, £22. | |
| General Nursing Institute. | |
| Grimsby Nursing Home. | |
| Hollond Institution, Nice. | |

### District Nurses.

| | |
|---|---|
| Bedwyn, Wilts., £25. | East London Nursing Society. |
| Berkhamsted. | Pontarfran, Brecon. |
| Bristol District Nurses' Society. | Rural Nursing Association. |
| Carwinion, £50. | Shanklin, £50. |

### Probationers.

| | |
|---|---|
| Bristol Hospital for Children. | Macclesfield Infirmary. |
| Brompton Cancer Hospital, £10. | Ophthalmic Hospital, £12. |
| Durham County Hospital. | Southport Massage Institution. |
| Kent Nursing Institution. | West Kent General Hospital. |
| Kilmarnock Infirmary. | Whitechapel Infirmary. |
| Liverpool Cancer Hospital. | Workhouse Infirmary Association. |

### Amusements and Relaxation.

### FOURTH QUARTERLY WORD COMPETITION
### Commenced Jan. 4th, 1890, ends March 29th, 1890.

Three Prizes of 15s., 10s., 5s., will be given for the largest number of words derived from the words set for dissection.

Proper names, abbreviations, foreign words, words of less than four letters, and repetitions are barred; plurals, and past and present participles of verbs, are allowed. Nuttall's *Standard* dictionary only to be used.

N.B.—Word dissections must be sent in WEEKLY, arranged alphabetically, with correct total affixed.

The word for dissection for this, the FOURTH week of the quarter, being "PENNY POST."

### Results of Second Week.

| Names. | Jan. 16th. | Totals. | Names. | Jan. 16th. | Totals. |
|---|---|---|---|---|---|
| Holland | 79 | 109 | Whitehoe | 80 | 110 |
| Red Cape | 27 | 62 | Nurse Clague | — | 40 |
| Nurse Marie | 66 | 93 | Lauriston | 88 | 136 |
| Garfield | 86 | 125 | B. E. T. | 61 | 77 |
| Midget | 81 | 121 | Jacko | 52 | 85 |
| E. M. S. | 70 | 110 | Lady Clara | 82 | 121 |
| Sister | 68 | 109 | Patience | 87 | 130 |
| Duchess | — | 39 | Ecila | 79 | 120 |
| Ellice | 76 | 122 | Maude | 86 | 126 |
| Reldas | 86 | 127 | Esperance | 85 | 126 |
| Jenny Wren | 82 | 120 | Ruby | 85 | 126 |
| Mopus | 85 | 122 | M. W. | 85 | 125 |
| Neptune | 78 | 118 | Crystal Palace | 86 | 86 |
| Qu'appelle | 85 | 126 | Jackanapes | 76 | 76 |

